# Systematic review and meta-analysis: proton pump inhibitors slightly decrease the severity of chronic cough

**DOI:** 10.1038/s41598-024-62640-9

**Published:** 2024-05-25

**Authors:** Diana-Elena Floria, Mahmoud Obeidat, Sarolta Beáta Kávási, Brigitta Teutsch, Dániel Sándor Veres, Krisztina Hagymási, Péter Hegyi, Vasile-Liviu Drug, Bálint Erőss

**Affiliations:** 1 Centre for Translational Medicine, Semmelweis University, Üllői Út 26, Budapest, 1085 Hungary; 2 Grigore T. Popa University of Medicine and Pharmacy Iaşi, University Street 16, Iaşi, 700115 Romania; 3 Institute for Translational Medicine, University of Pécs, Pécs, Hungary; 4 Department of Biophysics and Radiation Biology, Semmelweis University, Budapest, Hungary; 5 Institute of Pancreatic Diseases, Semmelweis University, Budapest, Hungary; 6 Department of Surgery, Toldy Ferenc Hospital, Cegléd, Hungary; 7 Department of Surgery, Transplantation and Gastroenterology, Faculty of Medicine, Semmelweis University, Budapest, Hungary; 8 Institute of Gastroenterology and Hepatology, Saint Spiridon Emergency Hospital Iaşi, Independence Boulevard 1, Iaşi, 700111 Romania

**Keywords:** Gastroenterology, Medical research

## Abstract

The Montreal consensus recognizes chronic cough as an extra-esophageal manifestation of gastroesophageal reflux disease. We performed a meta-analysis to assess the effects of acid-suppressive medications in adults with non-specific chronic cough. The protocol was registered on PROSPERO (CRD42022368769). Placebo-controlled randomized trials evaluating the impact of acid-suppressive medications on persistent cough were included. The systematic search was performed on the 1st of November 2022 in three databases. A random-effects model was used for the calculations. The effect size was the standardized mean difference (SMD) with 95% confidence interval (CI). A total number of 11 double-blinded placebo-controlled randomized trials were included in the meta-analysis. Data showed that compared to placebo, PPIs decreased the severity of cough (SMD 0.33; CI 0.05; 0.61). Therapeutic response was not different in patients with non-specific chronic cough only, compared to those with laryngopharyngeal reflux. Prolonged treatment durations did not result in greater symptomatic improvement, with SMD 0.33 (CI − 0.22; 0.88), 0.31 (CI − 1.74; 2.35), 0.32 (CI − 0.29; 0.93) and 0.34 (CI − 0.16; 0.85), following 4, 6, 8 and 12 weeks of treatment, respectively. The pooled analysis of the improvement in quality of life with PPIs found an SMD of 0.39 (CI − 0.51; 1.29). PPIs mildly decrease the severity of non-specific chronic cough, irrespective of treatment duration.

## Introduction

Non-specific chronic cough is commonly defined as a persistent cough for which a respiratory etiology or other known causes have been ruled out^1,2^. It has a high global prevalence, estimated to be around 9.6%^3^, with a significant negative impact on quality of life^4^.

The Montreal consensus recognizes this clinical entity as an extra-esophageal manifestation of gastroesophageal reflux disease (GERD)^5^. Epidemiologic studies report varying results, with GERD as the underlying disease in 10–41% of chronic cough patients^6^. Together with asthma and post-nasal drip syndrome, GERD represents one of the three leading causes of chronic cough^6^.

Given this association, acid-suppressive medications have been proposed as a therapeutic option in managing chronic cough after excluding other potential aetiologic factors. However, clinical studies investigating this intervention have yielded inconclusive results. A meta-analysis published in 2011 found insufficient data to support the beneficial effect of proton pump inhibitors (PPIs) in patients with chronic cough^1^. Even the most recent guidelines on this topic highlight the fact that the effect of acid-suppressive medication on extra-esophageal manifestations of GERD is still unclear^7–10^.

In the context of this knowledge gap, we aimed to assess the effects of acid-suppressive medications in adults with non-specific chronic cough, based on placebo-controlled randomized trials. Additionally, we set out to investigate the impact of these drugs on quality of life.

## Methods

This systematic review and meta-analysis was performed in compliance with the recommendations of the Preferred Reporting Items for Systematic Reviews and Meta-Analyses (PRISMA) 2020 Guideline^11^ and the Cochrane Handbook^12^. For the PRISMA Checklist, see Supplementary Material, page 16. The study protocol was prospectively registered on PROSPERO (CRD42022368769).

### Eligibility criteria

We used the population, intervention, comparator, outcome (PICO) framework to define the eligibility criteria. The population was comprised of adult patients with non-specific chronic cough, defined as a persistent cough for which a respiratory etiology or other known causes had been excluded. Any definition for the duration of chronic cough provided by the primary studies was accepted. The intervention was acid-suppressive medication, irrespective of dose, administration frequency, or treatment duration, with placebo as the comparator. The outcomes of interest were the impact on cough severity and quality of life, assessed by comparing the means of change in questionnaire scores. Only randomized controlled trials (RCTs) were included. Conference abstracts were considered eligible for inclusion if they reported data on the outcomes of interest. No restrictions were applied in terms of gender or ethnicity.

We excluded studies with inappropriate study design, pediatric population, and those which did not report cough severity or quality of life as an outcome.

### Information sources

The systematic search was conducted on the 1st of November 2022 in three medical databases: MEDLINE (via PubMed), Embase, and Cochrane Central Register of Controlled Trials (CENTRAL). No restrictions were applied. Additionally, backward and forward citation searching was conducted on the 29th of November 2022, to identify other potentially relevant publications^13^.

### Search strategy

The search key was comprised of three domains: cough, acid-suppressive medication, and the concept of randomization. For the detailed search key, see Supplementary Material page 3.

### Screening and selection process

The articles found through the systematic search were imported into a reference management program (EndNote, Clarivate Analytics Limited, London, UK). Removal of duplicate entries was performed automatically and manually by overlapping the authors, titles, and publication years. Two independent reviewers (D.E.I. and S.B.K.) carried out the screening and selection process, first by title and abstract, then by full text. On both levels of selection, Cohen's kappa coefficient (κ) was calculated as a measure of inter-reviewer agreement^14^. In case of conflict, a consensus was reached after discussing with a third investigator (M.O.).

### Data extraction

Relevant information from the included articles were independently extracted by two authors (D.E.I. and S.B.K.). A third reviewer (M.O.) resolved any disagreement. Data were manually collected and entered into an Excel table (Office 365, Microsoft, USA) as preparation for statistical analysis.

### Data items

The following information was extracted: name of the first author, year of publication, digital object identifier (DOI), country, study period and design, number of involved centers, detailed description of the study population, demographic information, details on the intervention (type of medication, dose, frequency of administration, duration of treatment), data on cough severity and quality of life (score values at baseline and at the end of treatment and/or the mean change), as well as details about the scale or scoring system used to assess them. For papers that reported information on the severity of cough or quality of life scores at multiple time points in the study, data was collected for all time points. Only data from the first period (before the switch to a different study arm) was used from cross-over studies, given the potential carry-over effect. Investigators attempted to contact the main authors of publications in order to obtain missing data elements; however, no additional information was retrieved. In order to assess the effects of acid-suppressive medication on cough severity in patients with laryngopharyngeal reflux, we only selected the symptom sub-scores pertaining to cough (not the overall scores).

### Risk of bias and quality of evidence assessment

Two reviewers (D.E.I. and S.B.K.) independently assessed the risk of bias using the ‘Revised Cochrane Risk-of-bias Tool for Randomized Trials’ (RoB 2)^15^. Any disagreements were resolved by discussion with a third reviewer (M.O.).

The tool is comprised of five domains through which bias may be introduced, assessing the randomization process (including allocation concealment and potential prediction), deviations from the intended interventions (including blinding of the participants and deliverers of the intervention, as well as the potential impact of the deviations on the outcomes), missing data (the availability of outcome data for most of the study participants, as well as missingness in results which may be related to their true value), measurements of the outcome (including methods used and blinding of outcome assessors), and potential selection of the reported result. Each one of these domains contains a series of signaling questions. Based on the answers of the assessors, final appraisals can be categorized as 'Low risk of bias,' 'Some concerns,' or 'High risk of bias.'

We used the Grading of Recommendations Assessment, Development and Evaluation (GRADE) approach^16^ and the GRADEpro tool (software) to evaluate the quality of evidence of our results.

### Statistical synthesis

Owing to varying scoring systems used across included studies, standardized mean difference (SMD, as Hedges’g^17^) with its 95% confidence interval (CI) was used for the effect size measure. For calculations, the mean, standard deviation (SD) or standard error of mean (SEM), and additionally the sample sizes were extracted, both for control and experimental groups. We reported the SMD of acid-suppressive medications compared to the placebo group using the estimated differences in cough severity and quality of life scores, from baseline to the end of treatment.

As considerable between-study heterogeneity was expected, a random-effects model was used in order to pool effect sizes.

The inverse variance weighting method was used for pooling SMDs. To estimate the heterogeneity variance measure τ^2^, the restricted maximum-likelihood estimator with the Q profile method for confidence interval^18^ was applied.

We used a Hartung–Knapp adjustment^19,20^ for CIs (if it provided a more conservative estimation compared to the classical approach, as recommended by Jackson et al.^21^, as hybrid method) and prediction intervals.

Additionally, between-study heterogeneity was described by means of Cochrane Q test, and the Higgins and Thompson’s I^2^ statistics^22^.

For subgroup analyses we employed a fixed-effects “plural” model (i.e. mixed-effects model). It was assumed that all subgroups shared a common τ^2^ value, as we did not anticipate differences in between-study heterogeneity in the subgroups. Moreover, the number of studies was relatively small in some subgroups. In order to assess the differences, the Cochrane Q test (an omnibus test) was used between subgroups^23^. The null hypothesis was rejected on a 5% significance level. Subgroup analysis was performed based on patient population, comparing participants presenting with chronic cough only and subjects with laryngopharyngeal reflux, which can present with accompanying chronic coughing.

Results were graphically summarized using forest plots and scatter plots (for the time dependency analysis).

Outlier and influence analyses were performed following the recommendations of Harrer et al.^23^ and Viechtbauer et al.^24^. For assessing the small study publication bias, visual inspection of funnel-plots was carried out. Additionally, we reported the Pustejovsky test *p*-value^25^, although it has limited diagnostic assessment below 10 studies. We performed influence analyses for the correlation coefficient assumption, used for estimating the differences from baseline to the end of treatment.

All statistical analyses were performed using the R software v4.3.0 (R Core Team, 2019, Vienna, Austria), using the meta (v6.5.0)^26^, metafor (v4.2.0)^27^ and dmetar (v0.0.9000)^28^ packages.

For additional details on the statistical synthesis, see the Supplementary Material, Statistical Analysis section.

### Ethical approval

No ethical approval was necessary to perform this systematic review and meta-analysis. Data was retrieved from articles previously published in peer-reviewed journals. There was no patient involvement in the study's design, conduct, or interpretation.

## Results

### Search and selection

**Figure 1:**
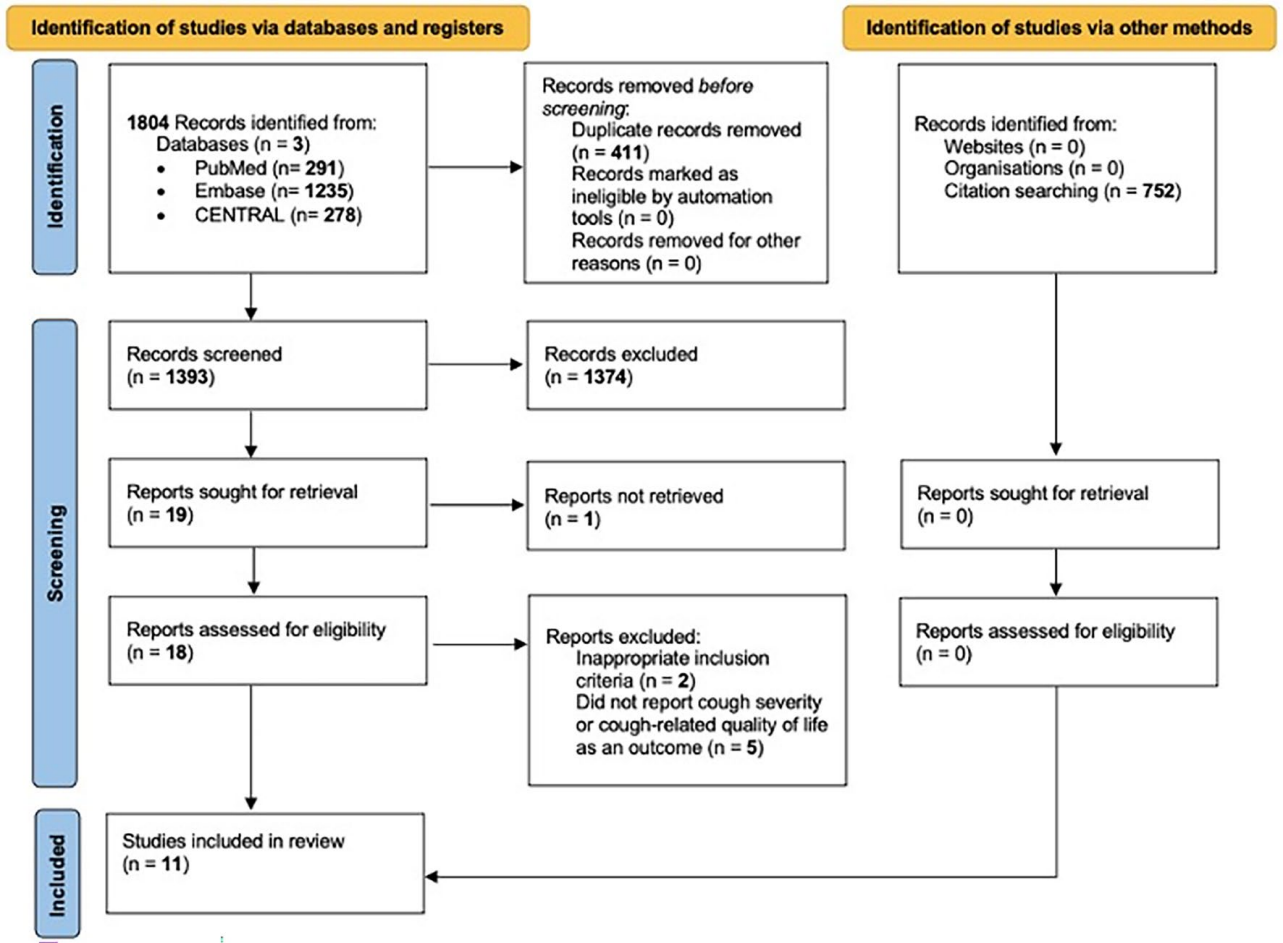
PRISMA 2020 flow chart detailing the search and selection process.

The systematic search yielded 1804 articles through the three medical databases, with 291 entries in MEDLINE (via PubMed), 1235 in Embase, and 278 in CENTRAL. Following duplicate removal, 1393 articles remained for the title and abstract selection. After the screening, 19 studies were sought for full-text evaluation (κ = 0.94), of which one record could not be retrieved. In total, 18 articles were assessed for eligibility, out of which seven were excluded^29–35^ (κ = 1). For the detailed reasons for exclusion, see Supplementary Material Table S1. 11 studies were finally included in the analysis^36–46^. The details of the search and selection process are presented in the PRISMA 2020 flow chart (Fig. 1).

### Basic characteristics of the selected studies

All the included articles were double-blinded placebo-controlled RCTs. Four studies were conducted in Europe, four in North America, two in Asia, and one in Australia. Regarding the study population, four articles included adults presenting with non-specific chronic cough only, while the other seven studies focused on patients with laryngopharyngeal reflux (which can manifest as persistent cough). All articles used PPIs with various doses, treatment durations, and frequencies of administration. Data on the impact on cough severity were reported in nine studies, while information on changes in the quality of life was found in five articles. In total, 612 participants were included in the analyses. The basic characteristics of the studies are presented in Table 1. For a detailed description of the patient population included in each study, see Supplementary Material Table S2.

**Table 1:** Basic characteristics of the included studies.

References	Country	Population	No of patients (female%)	Mean age (years)	Intervention	Outcome and scoring system (best to worst possible values)
Faruqi et al.^36^	UK	Chronic cough > 8 weeks	51 (64.0)	58.1	Esomeprazole 20 mg b.i.d. for 8 weeks	Cough severity: numerical scale for severity of cough (0–9) Quality of life: Leicester Cough Questionnaire (21–3)
Kiljander et al.^37^	Finland	Chronic cough > 8 weeks	29 (65.5)	49	Omeprazole 40 mg o.d. for 8 weeks	Cough severity: Weekly cough score (0–21)
Park et al.^39^	Korea	Chronic cough > 8 weeks	41 (51.9)	48.1	Esomeprazole 40 mg o.d./b.i.d. for 8 weeks	Cough severity: visual analogue scale (0–10) Quality of life: Leicester Cough Questionnaire (21–3)
Shaheen et al.^40^	USA	Chronic cough > 8 weeks	40 (78.0)	50	Esomeprazole 40 mg b.i.d. for 12 weeks	Cough Severity: cough severity score (0–4) Quality of life: cough-specific quality of life questionnaire (28–112)
Fass et al.^41^	USA	LPR	41 (41.5)	65.1	Esomeprazole 20 mg b.i.d. for 12 weeks	Quality of life: Laryngopharyngeal Reflux Health-Related Quality of Life Questionnaire—Sect. (0–36)
Havas et al.^42^	Australia	Posterior pharyngo-laryngitis	15 (53.3)	53.6	Lansoprazole 30 mg b.i.d. for 12 weeks	Cough severity: Cough score (0–7)
Lam et al.^43^	China	LPR	82 (72.0)	46.8	Rabeprazole 20 mg b.i.d. for 12 weeks	Cough severity: reflux symptom index—Troublesome or annoying cough Sect. (0–5)
Noordzij et al.^44^	USA	LPR	30 (46.6)	48.5	Omeprazole 40 mg b.i.d. for 8 weeks	Cough severity: cough symptom score (0–1400)
Reichel et al.^45^	Germany	LPR	62 (48.4)	48.7	Esomeprazole 20 mg b.i.d. for 12 weeks	Cough severity: reflux symptom index—Troublesome or annoying cough Sect. (0–5)
Steward et al.^46^	USA	LPR	42 (71.4)	49.3	Rabeprazole 20 mg b.i.d. for 8 weeks	Cough severity: dry cough symptom Questionnaire (0–8)
Wilson et al.^38^	UK	Persistent throat symptoms (including unexplained night-time chronic cough > 6 weeks)	346 (57.0)	52.2	Lansoprazole 30 mg b.i.d. for 16 weeks	Quality of Life: laryngopharyngeal reflux health-related quality of life questionnaire—cough Sect. (0–36)

(o.d.—*omne in die*, once daily; b.i.d.—*bis in die*, twice daily; LPR—laryngopharyngeal reflux).

### Mean change in cough severity

**Figure 2:**
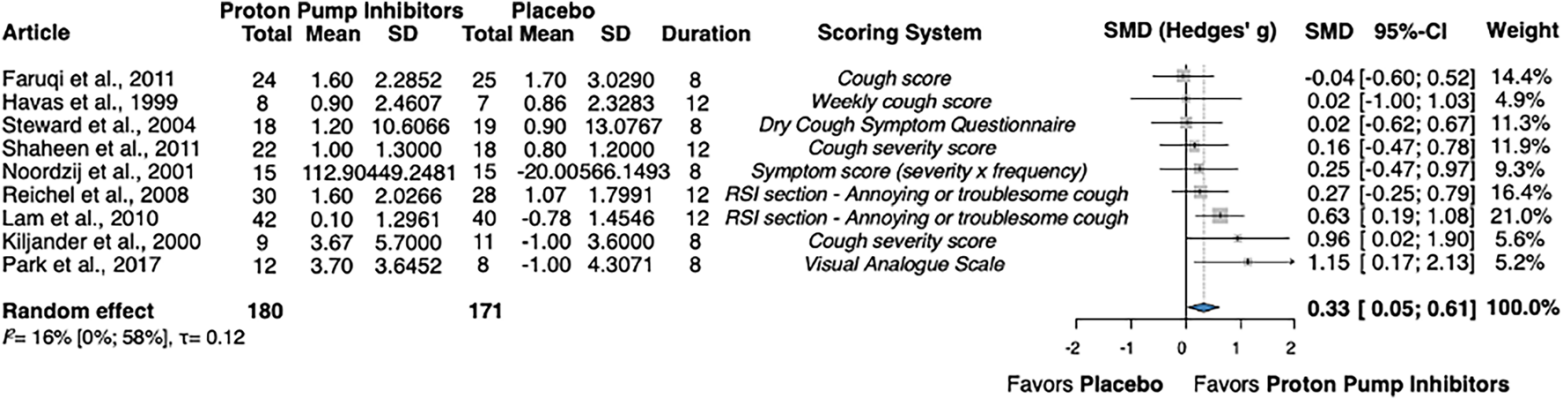
Forest plot demonstrating the change in mean cough severity in adults with chronic cough receiving treatment with proton pump inhibitors, compared to placebo. SMD, standardized mean difference; RSI, Reflux Symptom Index; CI, confidence interval; SD, standard deviation).

Nine studies reported data on the severity of cough, totaling 351 patients^36,37,39,40,42–46^. For articles that reported values at multiple time points in the study, data from the end of the treatment periods were used in the calculations. The analysis of the overall change in cough severity found an SMD of 0.33 (CI 0.05; 0.61), favoring PPIs (Fig. 2).

**Figure 3:**
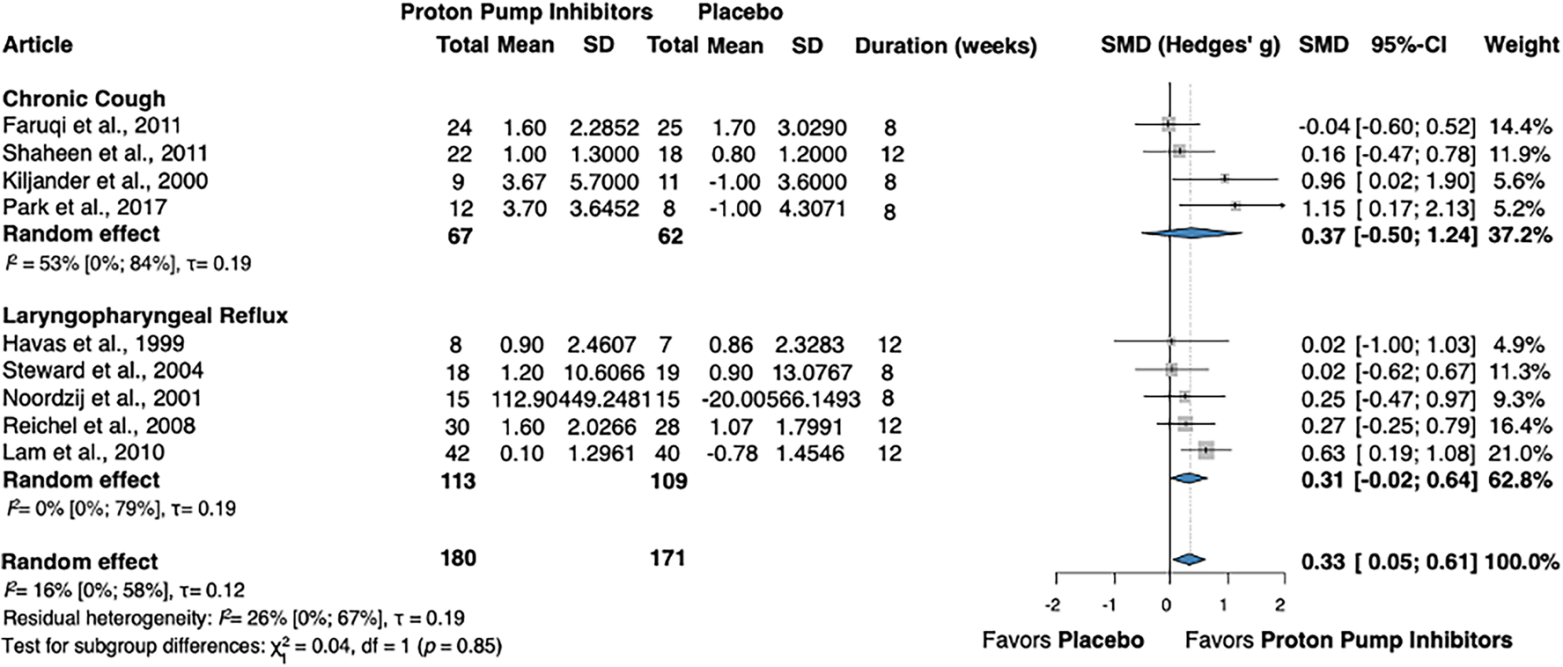
Forest plot demonstrating the mean change in cough severity in adults with chronic cough receiving treatment with proton pump inhibitors, compared to placebo – subgroup analysis based on patient population (chronic cough only, laryngopharyngeal reflux). SMD, standardized mean difference; CI, confidence interval; SD, standard deviation.

Subgroup analysis was performed based on patient population. Four articles included adults presenting with non-specific chronic cough only^36,37,39,40^, while the other five studies investigated patients with laryngopharyngeal reflux^42–46^. In the subgroup of almost 130 participants with persistent cough only, the SMD was 0.37 (CI − 0.50; 1.24). In the laryngopharyngeal reflux subgroup, which totaled more than 220 patients, the SMD was 0.31 (CI − 0.02; 0.64). There was no statistically significant difference between the two subgroups (Fig. 3).

The analysis of the change in cough severity in 67 patients with abnormal results on pH monitoring^37,40,44^ found an SMD of 0.49 (CI − 0.41; 1.39) (Supplementary Material Fig. S1. For a detailed description of the criteria used to establish abnormal reflux testing results in each of the included studies, see Supplementary Material Table S3.

**Figure 4:**
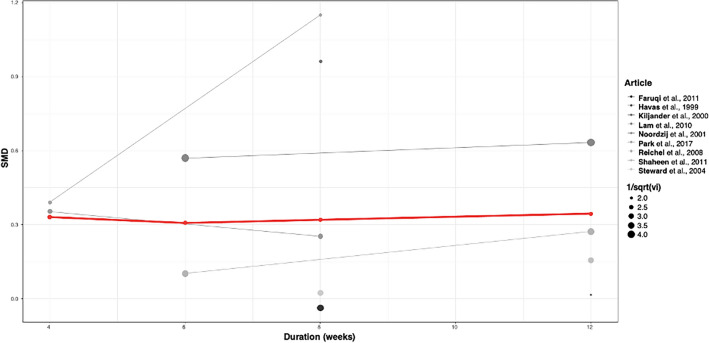
Time dependency analysis: improvement in mean cough severity according to the duration of acid-suppressive treatment with proton pump inhibitors (Y-axis shows the change in cough severity expressed as SMD—standardized mean difference; the X-axis shows the duration of acid-suppressive therapy in weeks. The point size represents the imprecision of articles expressed as the reciprocal of the sampling standard deviation).

The time dependency analysis found that longer treatment durations were not associated with more marked symptomatic improvement (Fig. 4). The estimated SMDs were 0.33 (CI − 0.22; 0.88), 0.31 (CI − 1.74; 2.35), 0.32 (CI − 0.29; 0.93), 0.34 (CI − 0.16; 0.85) following 4, 6, 8 and 12 weeks of acid-suppressive therapy, respectively.

### Mean change in the quality of life

**Figure 5:**
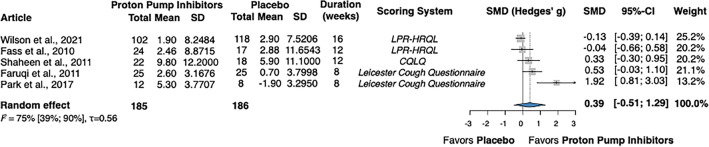
Forest plot demonstrating the change in mean quality of life in adults with chronic cough receiving treatment with proton pump inhibitors, compared to placebo. SMD, standardized mean difference; LPR-HRQL, laryngopharyngeal reflux-health-related quality of life; CQLQ, cough-related quality of life questionnaire; CI, confidence interval; SD, standard deviation.

Five studies investigated the impact of PPIs on the quality of life of adults with non-specific chronic cough compared to placebo, totaling 371 patients^36,38–41^. The pooled analysis found an SMD of 0.39 (CI − 0.51; 1.29) (Fig. 5).

### Heterogeneity

The heterogeneity was relatively low in the analysis of the overall change in cough severity (16%; CI 0%; 58%). In order to assess the robustness of conclusions concerning the pooled effect sizes, leave-one-out sensitivity analysis was performed. It found that the study by Lam et al.^43^ may have a more considerable impact on the estimation of the overall effect compared to the other articles. The omission of this study resulted in a change in SMD from 0.33 (CI 0.05; 0.61) to 0.24 (CI − 0.05; 0.54) (Supplementary Material Fig. S4).

In assessing the change in the cough-related quality of life following acid suppressive therapy compared to placebo, the heterogeneity was substantial (75%; CI 39%; 90%). According to the leave-one-out sensitivity analysis, the research of Park et al.^39^ may have a greater influence on the assessment of the total effect than the other publications. When this trial was omitted, the SMD changed from 0.39 (CI − 0.51; 1.29) to 0.11 (CI − 0.39; 0.62) (Supplementary Material Fig.S5).

### Risk of bias assessment

**Figure 6:**
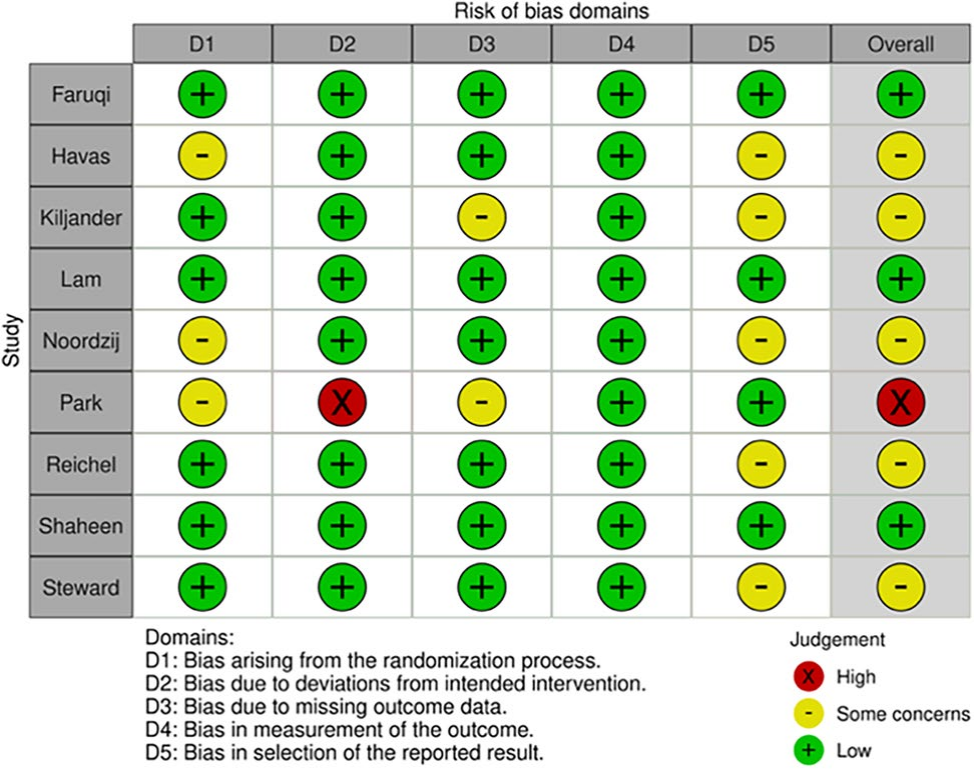
Risk of bias assessment results for cough severity in individual studies.

**Figure 7:**
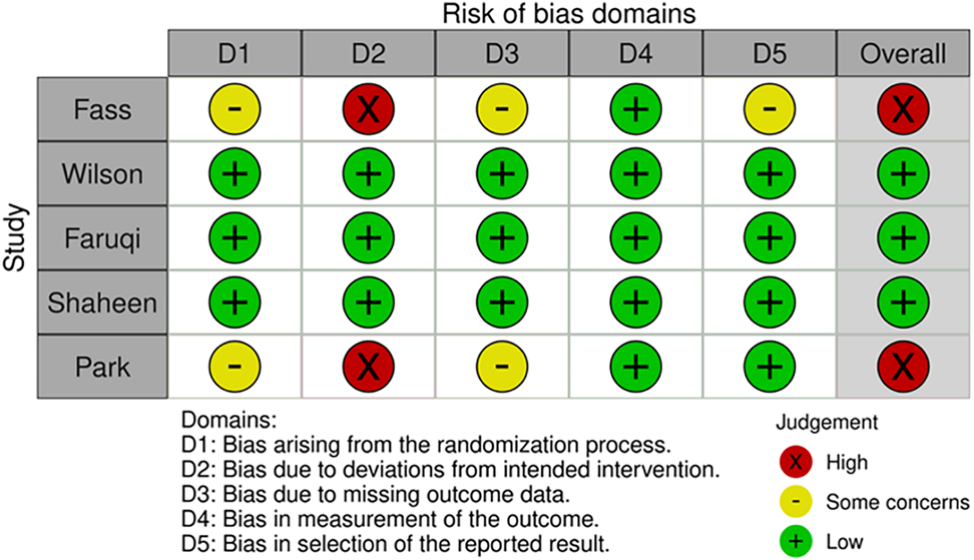
Risk of bias assessment results for quality of life in individual studies.

When the assessed outcome was cough severity, there were some concerns regarding the risk of bias in five of the articles^37,42,44–46^. Three were considered to be at low risk for bias^36,40,43^, while in one of the studies, the risk of bias was deemed high^39^ (Fig. 6, Supplementary Material Fig. S2). Regarding the quality of life, the risk of bias was considered low in three articles^36,38,40^ and high in the remaining two studies^39,41^ (Fig. 7, Supplementary Material Fig. S3).

### Certainty of evidence

When evaluating the effect of PPIs on cough severity, the level of evidence was considered low. The certainty of evidence was rated very low for the impact on quality of life. For a detailed assessment, see Supplementary Material Table S4.

## Discussion

Non-specific chronic cough, which may occur as an atypical manifestation of GERD, currently represents a challenging clinical entity from a therapeutic standpoint. Acid-suppressive drugs have been proposed as a potential treatment strategy, following the appropriate exclusion of other possible aetiologic factors. As clinical studies have yielded inconsistent results over the years, we set out to comprehensively evaluate their effects in adults presenting with non-specific chronic cough.

Based on the results of nine double-blinded, placebo-controlled RCTs, our study showed that acid-suppressive medications, namely PPIs, can slightly decrease cough severity. A previous meta-analysis published in 2011 by Chang et al.^1^ concluded that there was insufficient evidence to support the beneficial effect of PPIs in treating chronic cough. However, their analysis primarily focused on the proportion of patients who failed to respond to acid-suppressive therapy, with varying definitions of this outcome across included studies, and very limited data regarding the magnitude of the change in cough severity. Our study investigated the effect of PPIs by assessing the changes in cough scores from baseline to the end of treatment, compared to placebo. In line with the conclusion of the systematic review performed by Kahrillas et al.^2^ in 2013, we showed that acid-suppressive treatment may result in some degree of symptomatic improvement. It should be noted that while performing leave-one-out sensitivity analysis, the results were no longer statistically significant following the omission of the study by Lam et al^43^. This could be explained by the fact that this study had the largest sample size of all the included trials, and its exclusion led to wider confidence intervals of the pooled effect size.

We found that the effect of acid-suppressive medication on cough severity is similar in patients presenting with non-specific chronic cough only and those with laryngopharyngeal reflux, for which persistent coughing may be a symptom. The subgroup analysis showed that the symptomatic response in these two patient populations did not differ significantly. Noteworthy when this analysis was performed, the reduction in cough severity was not statistically significant in either of the two subgroups. This was probably because of the small number of included patients, resulting in wide confidence intervals.

Identifying subjects most likely to benefit from acid-suppressive treatment is essential. To that end, our results found that the improvement in cough scores with PPIs may be greater in subjects with chronic cough and abnormal reflux on pH monitoring. These findings are in line with the most recent recommendations of the American College of Gastroenterology and American Gastroenterological Association, suggesting that in patients presenting with suspected extra-digestive manifestations but no typical GERD symptoms, reflux testing off-PPI should be performed first^9,10^. It is essential to mention that only three of the included RCTs reported data on cough severity for subjects with objectively proven reflux^37,40,44^. Likely due to the small sample size, these clinically meaningful results did not reach statistical significance.

We also found that the magnitude of symptomatic improvement is likely unrelated to the administration duration. Based on currently available evidence, prolonged treatment durations do not seem to result in greater reductions in cough severity. Guidelines currently recommend that in patients with extra-esophageal manifestations and typical GERD symptoms, a trial of twice daily PPIs for up to 12 weeks may be advised^9,10^. However, data suggest that extended administration of acid-suppressive therapy will probably not result in a more pronounced symptomatic improvement compared to a treatment duration of 6–8 weeks.

Limited data was available regarding the effect of PPI therapy on cough-related quality of life. Our analysis identified the trial by Park et al.^39^ as a potential outlier, showing a noticeably larger change in the group receiving acid-suppressive medication. This study's exceptionally small sample size may account for the discrepancy, and the differences in baseline characteristics between the two study arms could further explain the inconsistency. The omission of this trial resulted in a much-decreased overall effect, suggesting that the improvement in perceived well-being following acid-suppressive therapy may be rather modest. These results did not reach statistical significance, possibly due to the small number of patients. Similar to the trend observed with cough severity, extended treatment durations were not associated with a greater amelioration in quality of life.

In the context of exploring the causal relationship between GERD and extraesophageal manifestations such as chronic cough, two principal mechanistic frameworks have been highlighted: the reflex theory and the reflux theory^47,48^. The reflux theory hypothesizes that the refluxate may ascend to the upper airways during reflux occurrences, inducing respiratory manifestations through micro-aspiration and direct stimulation. This concept offers insights into why patients receiving PPI therapy may continue to experience such symptoms despite non-acidic reflux. While acid-suppressive medication elevates the pH of the refluxate, it does not prevent the retrograde movement of the gastric contents.

It is also important to mention that while generally regarded as a safe medication, PPIs have undergone scrutiny for possible adverse effects associated with long-term use. For example, recent studies have observed an increased risk of developing community-acquired pneumonia, with up to two-fold higher odds in patients receiving PPI treatment compared to controls^49,50^. However, cautious interpretation of the data is advised, as these associations have been derived mostly from observational studies. One should bear in mind that causality cannot be established with certainty based on this type of studies, which are highly susceptible to bias^51,52^.

### Strengths and limitations

This study comprehensively assesses the effect of acid-suppressive medications, namely PPIs, in treating chronic cough. It focuses on the impact on cough severity and quality of life, yielding more precise insights using subgroup and time dependency analyses. Another strong point is the homogenous study design of the included articles, as we only selected double-blinded placebo-controlled RCTs. The rigorous methodological approach supports the validity of the results.

One of the main limitations of this study is the relatively small number of included patients. Given the limited number of studies, data had to be pooled irrespective of dose, administration frequency, or treatment duration. Also, the measurement of the outcomes was subjective, employing scales and questionnaires that varied widely across studies, some of which were not validated.

### Implications for practice and research

Translating scientific knowledge for community benefits is essential^53,54^. We believe that PPIs can have a beneficial effect in treating non-specific chronic cough in selected cases. Excluding other potential etiologies of cough, such as respiratory disorders or systemic diseases, is essential in these patients. In case of a lack of adequate response to initial treatment with PPIs, extending the duration of therapy will probably not result in marked symptomatic improvement.

From a research standpoint, we suggest that additional high-quality double-blinded placebo-controlled RCTs should be conducted, highlighting the need for appropriate sample sizes. Future clinical studies should seek to employ validated objective cough monitoring tools, such as the Leicester Cough Monitor and VitaloJAK^55,56^. Moreover, other potent acid-suppressive drugs, such as potassium-competitive acid blockers, may warrant further investigation in managing chronic cough.

## Conclusion

PPIs may marginally improve cough severity in some patients with non-specific chronic cough, which could be related to gastroesophageal reflux disease. Longer treatment durations are not associated with a more pronounced decrease in cough severity. Therefore, extending the duration of therapy is unlikely to result in marked symptomatic improvement.

### Supplementary Information

Supplementary Information.

## Data Availability

All the information in this study can be retrieved from the full-text articles in this systematic review and meta-analysis.

## References

[1] Chang AB, Lasserson TJ, Gaffney J, Connor FL, Garske LA (2011). Gastro-oesophageal reflux treatment for prolonged non-specific cough in children and adults. Cochrane Database Syst. Rev..

[2] Kahrilas PJ, Howden CW, Hughes N, Molloy-Bland M (2013). Response of chronic cough to acid-suppressive therapy in patients with gastroesophageal reflux disease. Chest.

[3] Song WJ (2015). The global epidemiology of chronic cough in adults: A systematic review and meta-analysis. Eur. Respir. J..

[4] Kang S-Y (2019). Impact of cough and unmet needs in chronic cough: A survey of patients in Korea. Lung.

[5] Vakil N (2006). The Montreal definition and classification of gastroesophageal reflux disease: A global evidence-based consensus. Am. J. Gastroenterol..

[6] Morice AH (2002). Epidemiology of cough. Pulm. Pharmacol. Ther..

[7] Kahrilas P (2016). Chronic cough due to gastroesophageal reflux in adults: CHEST guideline and expert panel report. Chest.

[8] Morice AH (2020). ERS guidelines on the diagnosis and treatment of chronic cough in adults and children. Eur. Respir. J..

[9] Katz PO (2022). ACG clinical guideline for the diagnosis and management of gastroesophageal reflux disease. Am. J. Gastroenterol..

[10] Chen JW, Vela MF, Peterson KA, Carlson DA (2023). AGA clinical practice update on the diagnosis and management of extraesophageal gastroesophageal reflux disease: Expert review. Clin. Gastroenterol. Hepatol. Off. Clin. Pract. J. Am. Gastroenterol. Assoc..

[11] Page MJ (2021). The PRISMA 2020 statement: An updated guideline for reporting systematic reviews. BMJ.

[12] Higgins J (2022). Cochrane Handbook for Systematic Reviews of Interventions version 6.3 (updated February 2022).

[13] Haddaway, N. R., Grainger, M. J. & Gray, C. T. citationchaser: An R package and Shiny app for forward and backward citations chasing in academic searching. 10.5281/zenodo.4543513 (2021)

[14] Cohen J (1960). A coefficient of agreement for nominal scales. Educ. Psychol. Meas..

[15] Sterne JAC (2019). RoB 2: A revised tool for assessing risk of bias in randomised trials. BMJ.

[16] Guyatt G (2011). GRADE guidelines: 1. Introduction-GRADE evidence profiles and summary of findings tables. J. Clin. Epidemiol..

[17] Hedges LV (1981). Distribution theory for glass’s estimator of effect size and related estimators. J. Educ. Stat..

[18] Veroniki AA (2016). Methods to estimate the between-study variance and its uncertainty in meta-analysis. Res. Synth. Methods.

[19] Knapp G, Hartung J (2003). Improved tests for a random effects meta-regression with a single covariate. Stat. Med..

[20] IntHout J, Ioannidis JPA, Borm GF (2014). The Hartung–Knapp–Sidik–Jonkman method for random effects meta-analysis is straightforward and considerably outperforms the standard DerSimonian-Laird method. BMC Med. Res. Methodol..

[21] Jackson D, Law M, Rücker G, Schwarzer G (2017). The Hartung–Knapp modification for random-effects meta-analysis: A useful refinement but are there any residual concerns?. Stat. Med..

[22] Higgins JPT, Thompson SG (2002). Quantifying heterogeneity in a meta-analysis. Stat. Med..

[23] Harrer M, Cuijpers P, Toshi F, Ebert DD (2021). Doing Meta-Analysis with R: A Hands-on Guide.

[24] Viechtbauer W, Cheung MW-L (2010). Outlier and influence diagnostics for meta-analysis. Res. Synth. Methods.

[25] Pustejovsky, J. clubSandwich: Cluster-robust (Sandwich) variance estimators with small-sample corrections. (2022).

[26] Schwarzer, G. Meta: General package for meta-analysis. (2022).

[27] Viechtbauer, W. Metafor: Meta-analysis package for r. (2023).

[28] Pim, C., Furukawa, T. & Ebert, D. D. Dmetar: Companion r package for the guide doing meta-analysis in r (2022).

[29] Anzić SA (2018). Eight weeks of omeprazole 20 mg significantly reduces both laryngopharyngeal reflux and comorbid chronic rhinosinusitis signs and symptoms: Randomised, double-blind, placebo-controlled trial. Clin. Otolaryngol..

[30] El-Serag HB (2001). Lansoprazole treatment of patients with chronic idiopathic laryngitis: A placebo-controlled trial. Am. J. Gastroenterol..

[31] Eherer AJ (2003). Effect of pantoprazole on the course of reflux-associated laryngitis: A placebo-controlled double-blind crossover study. Scand. J. Gastroenterol..

[32] Ing A (1997). Chronic cough. Respirology.

[33] Ours TM, Kavuru MS, Schilz RJ, Richter JE (1999). A prospective evaluation of esophageal testing and a double-blind, randomized study of omeprazole in a diagnostic and therapeutic algorithm for chronic cough. Am. J. Gastroenterol..

[34] Vaezi MF (2006). Treatment of chronic posterior laryngitis with esomeprazole. Laryngoscope.

[35] Wo JM (2006). Double-blind, placebo-controlled trial with single-dose pantoprazole for laryngopharyngeal reflux. Am. J. Gastroenterol..

[36] Faruqi S (2011). Chronic cough and esomeprazole: A double-blind placebo-controlled parallel study. Respirology.

[37] Kiljander TO, Salomaa ERM, Hietanen EK, Terho EO (2000). Chronic cough and gastro-oesophageal reflux: A double-blind placebo-controlled study with omeprazole. Eur. Respir. J..

[38] Wilson JA (2021). Lansoprazole for persistent throat symptoms in secondary care: The TOPPITS RCT. Health Technol. Assess..

[39] Park HJ (2017). Effectiveness of proton pump inhibitor in unexplained chronic cough. PLoS One.

[40] Shaheen NJ (2011). Randomised clinical trial: High-dose acid suppression for chronic cough—A double-blind, placebo-controlled study. Aliment. Pharmacol. Ther..

[41] Fass R (2010). The effect of esomeprazole 20 mg twice daily on acoustic and perception parameters of the voice in laryngopharyngeal reflux. Neurogastroenterol. Motil..

[42] Havas T (1999). Posterior pharyngolaryngitis. Double-blind randomised placebo-controlled trial of proton pump inhibitor therapy. Aust. J. Otolaryngol..

[43] Lam PKY (2010). Rabeprazole is effective in treating laryngopharyngeal reflux in a randomized placebo-controlled trial. Clin. Gastroenterol. Hepatol..

[44] Noordzij JP (2001). Evaluation of omeprazole in the treatment of reflux laryngitis: A prospective, placebo-controlled, randomized, double-blind study. Laryngoscope.

[45] Reichel O, Dressel H, Wiederänders K, Issing WJ (2008). Double-blind, placebo-controlled trial with esomeprazole for symptoms and signs associated with laryngopharyngeal reflux. Otolaryngol. Head Neck Surg..

[46] Steward DL (2004). Proton pump inhibitor therapy for chronic laryngo-pharyngitis: A randomized placebo-control trial. Otolaryngol. Head Neck Surg..

[47] Iov, D.-E., Erőss, B., Obeidat, M., Drug, V. L. & Kávási, S. B. Therapeutic effects of acid-suppressive medications in adults with non-specific chronic cough: A systematic review and meta-analysis. *PROSPERO* CRD42022368769 (2022).

[48] Wu J, Ma Y, Chen Y (2022). GERD-related chronic cough: Possible mechanism, diagnosis and treatment. Front. Physiol..

[49] Lambert AA (2015). Risk of community-acquired pneumonia with outpatient proton-pump inhibitor therapy: A systematic review and meta-analysis. PLoS One.

[50] Nguyen PA (2020). Meta-analysis of proton pump inhibitors induced risk of community-acquired pneumonia. Int. J. Qual. Heal. Care.

[51] Horwitz RI, Feinstein AR (1980). The problem of ‘protopathic bias’ in case-control studies. Am. J. Med..

[52] Bosco JLF (2010). A most stubborn bias: No adjustment method fully resolves confounding by indication in observational studies. J. Clin. Epidemiol..

[53] Hegyi P (2020). Academia Europaea position paper on translational medicine: The cycle model for translating scientific results into community benefits. J. Clin. Med..

[54] Hegyi P, Erőss B, Izbéki F, Párniczky A, Szentesi A (2021). Accelerating the translational medicine cycle: The Academia Europaea pilot. Nature medicine.

[55] Birring SS (2008). The Leicester cough monitor: Preliminary validation of an automated cough detection system in chronic cough. Eur. Respir. J..

[56] Smith JA (2021). Performance of a digital signal processing algorithm for the accurate quantification of cough frequency. Eur. Respir. J..

