# Supplementary material for: Systematic review and meta-analysis: proton pump inhibitors slightly decrease the severity of chronic cough
Source: Sci Rep. 2024 May 25;14:11956. doi: 10.1038/s41598-024-62640-9 (PMC11127940; doi:10.1038/s41598-024-62640-9)
Supplement: Supplementary file 1 — Supplementary Information. [file 41598_2024_62640_MOESM1_ESM.docx]

**Systematic Review and Meta-analysis: Proton Pump Inhibitors Slightly Decrease the Severity of Chronic Cough**

**Supplementary Material**

**Figure and Table Legends**

**Detailed Search Key**

**Table S1.** Reasons for exclusion following full-text assessment.

**Table S2.** Detailed description of the population in the included studies.

**Figure S1.** Mean change in cough severity in the subset of patients with chronic cough and abnormal pH studies.

**Figure S2.** Overall risk of bias assessment results for cough severity for each domain.

**Figure S3.** Overall risk of bias assessment results for quality of life for each domain.

**Figure S4.** Leave-one-out sensitivity analysis for the change in mean cough severity.

**Figure S5.** Leave-one-out sensitivity analysis for the change in mean quality of life.

**Table S3.** Detailed assessment of the certainty of evidence.

**Statistical analysis**

**Search Key:**

**Embase**

(cough or (laryngopharyngeal reflux) OR (LPR) or (reflux laryngitis)) AND ((acid suppress*) or (pump inhibitor) OR (ppi) OR rabeprazole OR lansoprazole OR dexlansoprazole OR pantoprazole OR esomeprazole OR omeprazole OR tenatoprazole OR (h2 receptor antagonist) OR (histamine receptor antagonist) OR (h2 blocker) OR (H2RA) OR ranitidine OR famotidine OR nizatidine or cimetidine OR revaprazan OR vonoprazan OR tegoprazan) AND (random* or RCT)

**PubMed**

(cough or (laryngopharyngeal reflux) OR (LPR) or (reflux laryngitis)) AND ((acid suppress*) or (pump inhibitor) OR (ppi) OR rabeprazole OR lansoprazole OR dexlansoprazole OR pantoprazole OR esomeprazole OR omeprazole OR tenatoprazole OR (h2 receptor antagonist) OR (histamine receptor antagonist) OR (h2 blocker) OR (H2RA) OR ranitidine OR famotidine OR nizatidine or cimetidine OR revaprazan OR vonoprazan OR tegoprazan) AND (random* or RCT)

**CENTRAL**

(cough or (laryngopharyngeal reflux) OR (LPR) or (reflux laryngitis)) AND ((acid suppress*) or (pump inhibitor) OR (ppi) OR rabeprazole OR lansoprazole OR dexlansoprazole OR pantoprazole OR esomeprazole OR omeprazole OR tenatoprazole OR (h2 receptor antagonist) OR (histamine receptor antagonist) OR (h2 blocker) OR (H2RA) OR ranitidine OR famotidine OR nizatidine or cimetidine OR revaprazan OR vonoprazan OR tegoprazan) AND (random* or RCT)

**Table S1.** Table showing the reasons for exclusion following full-text assessment.

| **Author, year** | **Title** | **Reason for exclusion** |
| --- | --- | --- |
| Anzić et al, 2018 | Eight weeks of omeprazole 20 mg significantly reduces both laryngopharyngeal reflux and comorbid chronic rhinosinusitis signs and symptoms: randomized, double-blind, placebo-controlled trial | Inappropriate population (Comorbid chronic rhinosinusitis) |
| Eherer et al, 2003 | Effect of Pantoprazole on the Course of Reflux-Associated Laryngitis: a Placebo-Controlled Double-Blind Crossover Study | Did not report cough severity and/or quality of life as an outcome. |
| El-Serag et al, 2001 | Lansoprazole Treatment of Patients With Chronic Idiopathic Laryngitis: A Placebo-Controlled Trial | Did not report cough severity and/or quality of life as an outcome. |
| Ing et al, 1997 | Chronic cough | Inappropriate population (Recent upper respiratory tract infection in most subjects) |
| Ours et al, 1999 | A Prospective Evaluation of Esophageal Testing and a Double-Blind, Randomized Study of Omeprazole in a Diagnostic and Therapeutic Algorithm for Chronic Cough | Did not report cough severity and/or quality of life as an outcome. |
| Vaezi et al, 2006 | Treatment of Chronic Posterior Laryngitis With Esomeprazole | Did not report cough severity and/or quality of life as an outcome. |
| Wo et al, 2006 | Double-Blind, Placebo-Controlled Trial with Single-Dose Pantoprazole for Laryngopharyngeal Reflux | Did not report cough severity and/or quality of life as an outcome. |

**Table S2.** Table showing the detailed description of the population in the included studies.

| **Author, Year** | **Country** | **Population** |
| --- | --- | --- |
| Faruqi et al, 2011 | UK | **Chronic cough** >8 weeks, with a cough score ≥3 (non-smokers, normal chest radiograph, no obvious lung disease, no recent respiratory tract infection, no angiotensin-converting enzyme inhibitors, no anti-acid medication); clinical features consistent with reflux-related cough (cough on phonation or on bending in association with food and eating). |
| Kiljander et al, 2000 | Finland | **Chronic cough** >8 weeks (non-smokers, normal chest radiograph, no rhinitis or sinusitis, no post-nasal drip syndrome, no asthma or chronic bronchitis, no angiotensin-converting enzyme inhibitors) and abnormal pH study. |
| Park et al, 2017 | Korea | **Chronic cough** >8 weeks (non-smokers, no post-nasal drip syndrome, no asthma-related cough syndrome, no underlying pulmonary disease, no previous failure to PPI therapy or anti-reflux procedures, no Barrett’s esophagus or digestive tumors, no recent upper respiratory infection, no current use of PPI, H2 blocker, beta-blocker, angiotensin-converting enzyme inhibitors, corticosteroid, methylxanthine, or anticholinergics). |
| Shaheen et al, 2011 | USA | **Chronic cough** >8 weeks (non-smokers, normal chest radiograph, no post-nasal drip syndrome, no reported heartburn symptoms more than two times/month, no failure to previous PPI treatment, no antireflux procedure, no previous aerodigestive malignancy or Barrett’s esophagus, no recent upper respiratory infection, no PPI, H2 blocker, beta-blocker, angiotensin-converting enzyme inhibitors, corticosteroid, methylxanthine, inhaled beta-agonist, anti-inflammatory agent, or anticholinesterase drug). |
| Fass et al, 2010 | USA | **Laryngopharyngeal reflux** diagnosed by an otolaryngologist based on laryngeal symptoms and laryngoscopy (no antireflux treatment, no prior gastroesophageal surgery, no endotracheal intubation within the previous 3 months, no oropharyngeal or laryngeal cancer, no severe comorbidity, no diabetes mellitus, no scleroderma, no gastroparesis, no active peptic ulcer disease, no current treatment with prokinetics or narcotics). |
| Havas et al, 1999 | Australia | **Posterior pharyngo-laryngitis** diagnosed by an otolaryngologist (no significant neurological disorders, no chronic airflow limitation, no prior anti-secretory medication, no severe esophagitis on endoscopy, no professional voice users). |
| Lam et al, 2010 | China | **Laryngopharyngeal reflux** diagnosed by an otolaryngologist based on laryngeal symptoms and laryngoscopy (no recent upper respiratory tract infection, no allergic causes of laryngitis, no identifiable laryngeal pathology other than suspected LPR, no previous radiotherapy, no gastroesophageal surgery, no recent acid-suppressive therapy). |
| Noordzij et al, 2001 | USA | **Laryngopharyngeal reflux** diagnosed by an otolaryngologist based on laryngeal symptoms (including chronic cough>12 weeks), laryngoscopy and >4 episodes of LPR on 24-hour dual-channel pH probe testing (no viral or bacterial laryngitis, no laryngeal cancer, no benign vocal fold lesions, no history of seasonal allergies, no occupational exposures). |
| Reichel et al, 2008 | Germany | **Laryngopharyngeal reflux** diagnosed by an otolaryngologist based on laryngeal symptoms (including chronic cough) and laryngoscopy (no recent treatment with PPI/other anti-reflux medication, no history of laryngeal malignancy or gastrointestinal surgery, no need for continuous therapy with warfarin, coumarin, or acetylsalicylic acid). |
| Steward et al, 2004 | USA | **Laryngopharyngeal reflux** diagnosed by an otolaryngologist based on laryngeal symptoms (including chronic cough >4weeks) and laryngoscopy (no previous surgery for GERD, no current gastrostomy tube or tracheotomy tube, no endotracheal tube intubation within previous 2 months, no history of hypersecretory disorder, no recent use of a PPI or H2 blocker, no current systemic steroid therapy, no history of laryngeal or hypopharyngeal neoplasm, no previous radiation therapy to neck, no diagnosis of vocal cord paralysis, no active granulomatous disease requiring systemic therapy, no suspicion for laryngeal neoplasm requiring biopsy for diagnosis). |
| Wilson et al, 2021 | UK | **Persistent throat symptoms** (including unexplained night-time chronic cough >6weeks) (no observed endoscopic laryngopharyngeal pathology that would typically require specific surgical intervention or investigations, no current or prior malignancy of the head and neck or oesophagus, no performing voice users, no current or recent use of acid suppressants/acid neutralisers/alginates, no severe hepatic dysfunction, no need for treatment with: warfarin, phenytoin, digoxin, ciclosporin, methotrexate, erlotinib, lapatinib, tacrolimus, sucralfate, citalopram, escitalopram, fluvoxamine, St John’s wort, clozapine, ulipristal acetate, cilostazol or systemic antifungals, no human immunodeficiency virus-positive patients/patients taking antiviral medications, no other investigational study drugs within the preceding 30 days.) |

*GERD: gastroesophageal reflux disease; LPR: laryngopharyngeal reflux; PPI: proton pump inhibitor; H2 blocker: histamine H2-receptor blocker*

***Table S3.*** Table showing the detailed definition of abnormal pH testing results as reported in the included studies.

| **Author, year** | **Definition** |
| --- | --- |
| Kiljander et al., 2000 | pH Monitoring was considered to be abnormal if total time pH <4 was over 4.5% or the DeMeester score was over 14.7. |
| Shaheen et al., 2011 | A DeMeester score of greater than 14.7 was categorized as “high distal esophageal acid” and a score of 14.7 or less was classified as “low distal esophageal acid”. |
| Noordzij et al., 2001 | More than four episodes of laryngopharyngeal reflux (1 cm above the upper esophageal sphincter) per 24 hours to be included. An episode of laryngopharyngeal reflux was defined as a drop in pH below 4 or a 3-point drop in pH (resulting in a pH of less than 5). This episode had to occur with a simultaneous esophageal drop in pH below 4. |


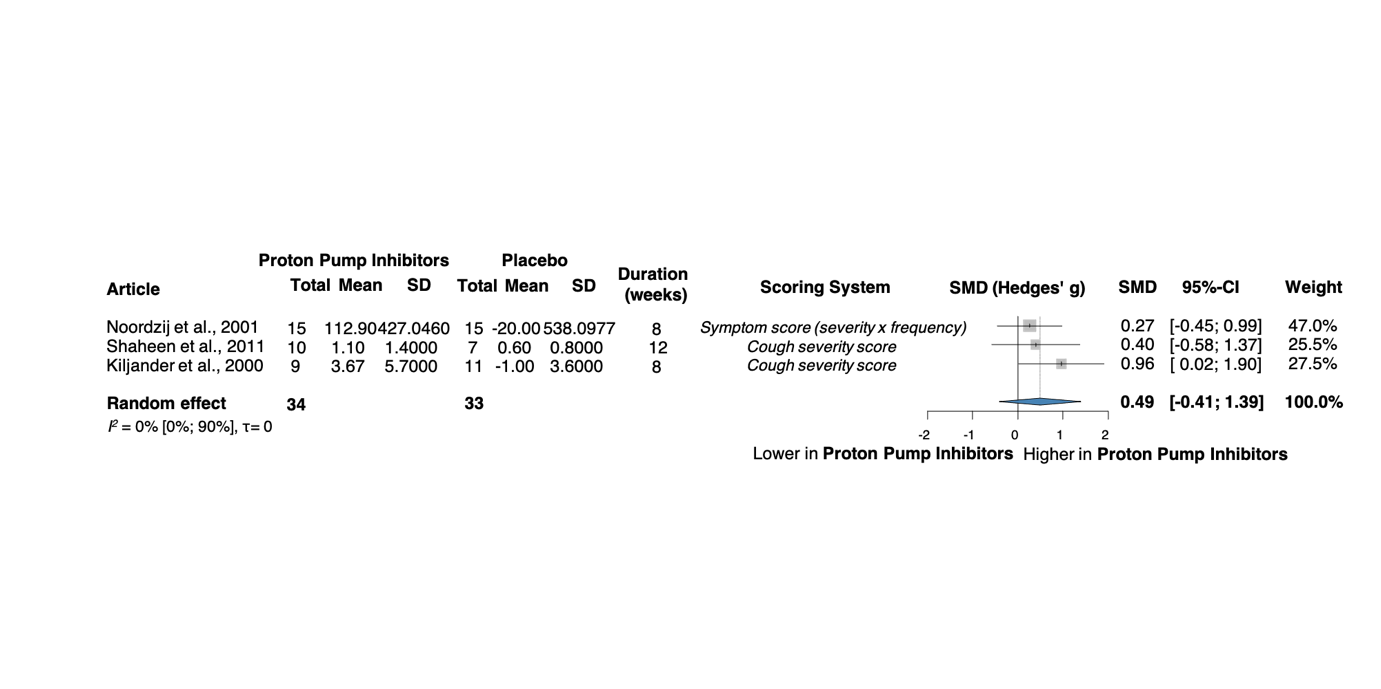


**Figure S1.** Forest plot demonstrating the change in mean cough severity in the subset of patients with chronic cough and abnormal pH studies (*SMD, standardized mean difference; CI, confidence interval; SD, standard deviation*)


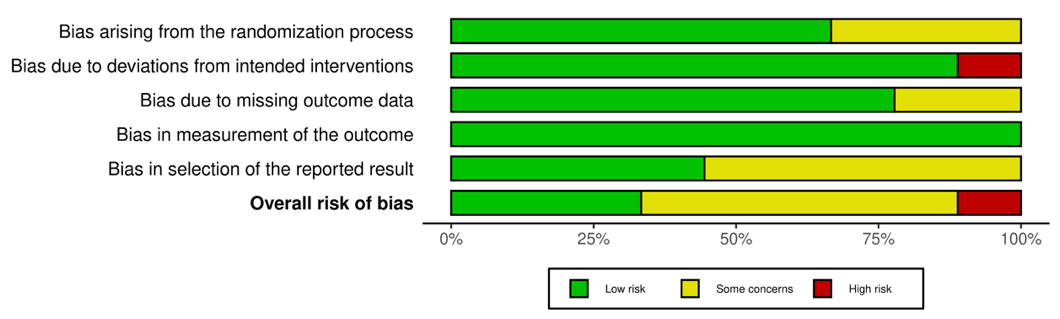


**Figure S2.** Overall risk of bias assessment results for cough severity for each domain.


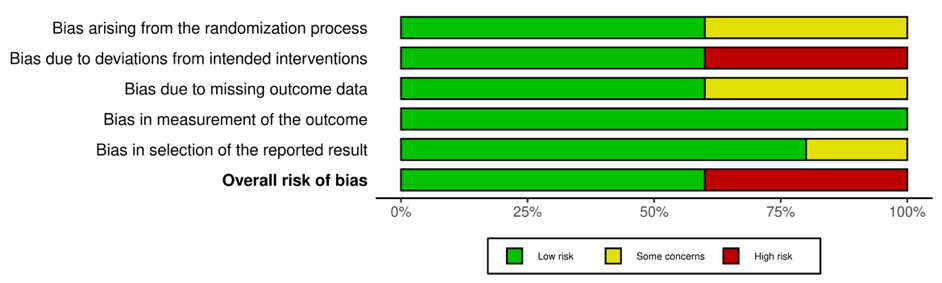


**Figure S3.** Overall risk of bias assessment results for quality of life for each domain.


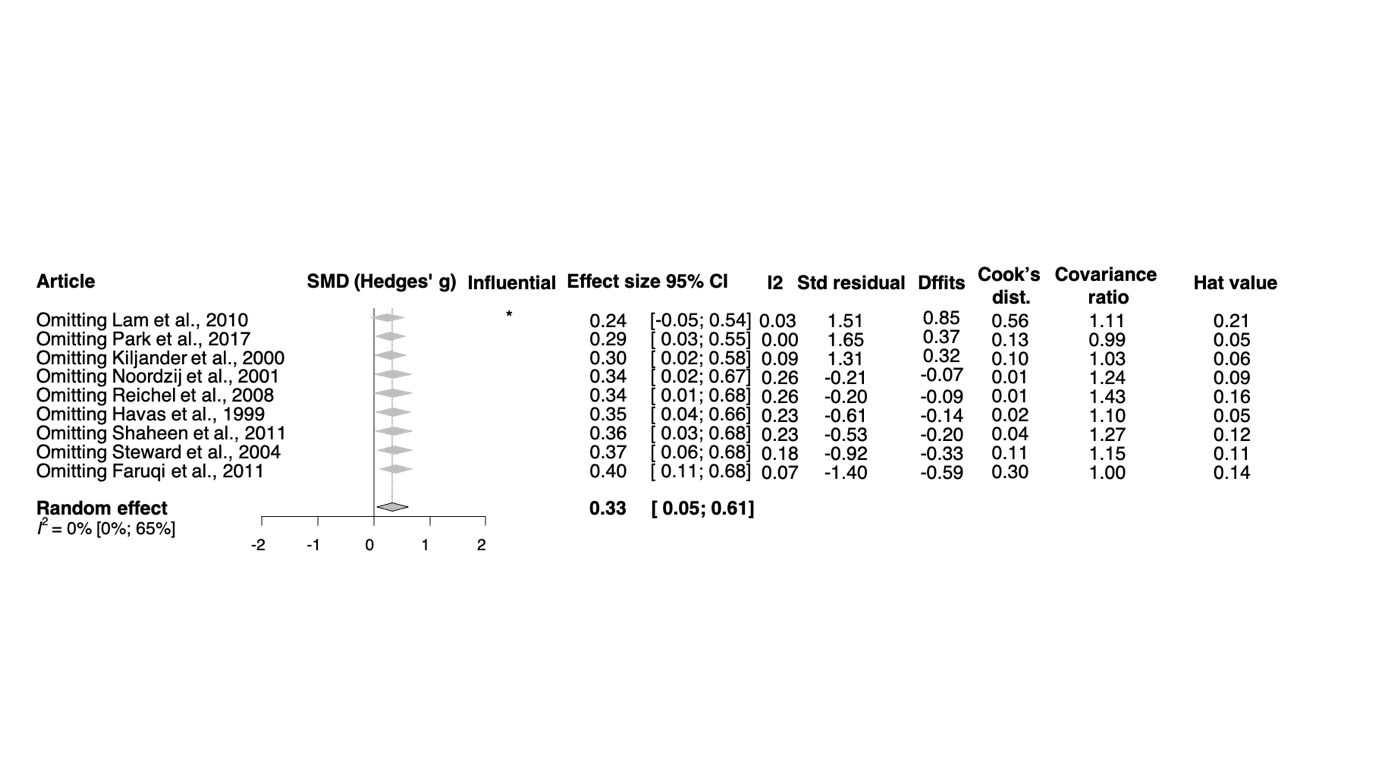


**Figure S6.** Leave-one-out sensitivity analysis for the change in mean cough severity (*SMD, standardized mean difference;* *CI, confidence interval)*


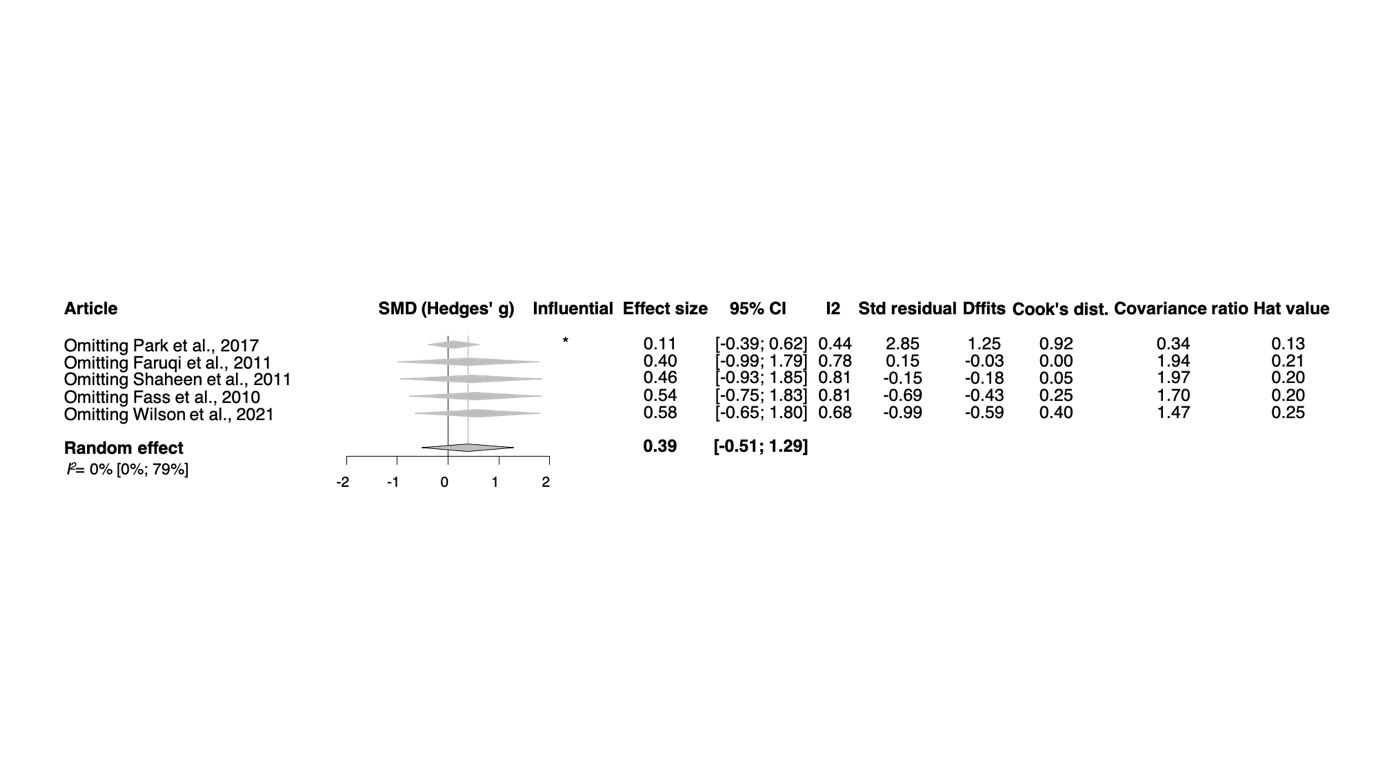


**Figure S7.** Leave-one-out sensitivity analysis for the change in mean quality of life (*SMD, standardized mean difference;* *CI, confidence interval).*

**Table S4.** Detailed assessment of the certainty of evidence.

| **Certainty assessment** | | | | | | | **№ of patients** | | **Effect** | | **Certainty** | **Importance** |
| --- | --- | --- | --- | --- | --- | --- | --- | --- | --- | --- | --- | --- |
| **№ of studies** | **Study design** | **Risk of bias** | **Inconsistency** | **Indirectness** | **Imprecision** | **Other considerations** | **PPIs** | **Placebo** | **Relative (95% CI)** | **Absolute (95% CI)** |  |  |
| **Change in Cough Severity** | | | | | | | | | | | | |
| 9 | randomised trials | serious^a^ | not serious | not serious | serious^b^ | none | 180 | 171 | - | SMD **0.33 SD higher** (0.05 higher to 0.61 higher) | ⨁⨁◯◯ Low | IMPORTANT |
| **Change in Quality of Life** | | | | | | | | | | | | |
| 5 | randomised trials | serious^c^ | serious^d^ | not serious | serious^e^ | none | 185 | 186 | - | SMD **0.39 SD higher** (0.51 lower to 1.29 higher) | ⨁◯◯◯ Very low | IMPORTANT |

**CI:** confidence interval; **SMD:** standardised mean difference

#### Explanations

a. There were some concerns for risk of bias in almost half of the included studies. These mostly arose from the lack of a pre-published protocol. The authors decided to downgrade the level of evidence by one level.

b. Given the fact that the pooled sample size was less than 400, the authors decided to rate down for imprecision.

c. The risk of bias was high in two out of five studies (arising from the lack of details about the randomisation process, about the flow of patients - only include details about the ones that completed the treatment, large numbers of patients lost to follow-up - which resulted in differences in baseline characteristics between the two study arms). For this reason, the authors decided to downgrade the level of evidence.

d. The pooled analysis of the effect of proton pump inhibitors on quality of life showed substantial heterogeneity (I2=75%; CI: 39%; 90%). Some of the studies showed larger improvement in cough-related quality of life with placebo, while others greatly favoured proton pump inhibitors. Therefore, the authors chose to downgrade the level of evidence.

e. The pooled sample size was relatively small, less than 400. For this reason, the level of evidence was downgraded by one level.

**Statistical analysis**

In order to calculate the pooled difference, the sample size, mean, standard deviation (SD) or standard error of mean (SEM) were extracted from each study, both for control and experimental groups. Where available, the mean and SD values were used for the calculation. If SD was not given, but instead the SEM was available, the SD was calculated as SEM multiplied by the square root of the sample size. If instead of the mean, SD or SEM, the quartiles were given, Luo and Shi methods^1,2^ (as implemented in the used meta R package) were used for estimating the mean and SD from the quartiles. Although, based on other publications about these outcomes and the data in the included studies, we could assume that the distribution of this variable is not relevantly different from a normal or log-normal distribution, therefore this estimation might give only a small bias.

As the outcomes were measured using various scales across studies, we used standardised mean difference (SMD)^3^, with its corresponding 95% confidence interval (CI) for the effect size measure. From the given or estimated corresponding sample sizes, means and SDs, we calculated the SMD. The SMD was calculated as bias corrected Hedges’ g based on Hedges et al.^3^, as implemented in the *metafor* R package *escalc* function with argument *measure=“SMD”*.

Kiljander et al.^4^ performed a cross-over study, from which we only used the values from the first study period (i.e. before the cross-over). From this article we could extract individual patient data (IPT) from figures, which were also used in this analysis.

Several studies reported the observed values at baseline and at the end of the treatment period, but the mean change was not provided. On the other hand, some articles reported only the mean change, without presenting the baseline and after treatment values. Therefore, these values could not be pooled together directly. In this situation, **four analyses (*see below*)** were performed in order to estimate the effect and its significance.

**1.** As we only included randomized controlled trials, we assumed that the baseline values were equal in the experimental and control group. Therefore, the effect could be expressed by using only the reported after-treatment values, for the articles where these are reported (therefore, no assumptions or estimations were necessary). (Not reported in the article)

**2.** Where mean change values were directly reported, they could be used for pooling. (Not reported in the article)

**3.** In order to pool together results from the included studies, we needed to estimate the means and SD of the changes from baseline to after-treatment, if these were not reported. For this, we estimated the correlation coefficient (R) between baseline and after-treatment values. The estimation of R was based on the reported or calculated SD of the change if the SD for baseline and after-treatment values were provided. Based on this, the correlation coefficient was calculable; two studies reported these data: Kiljander et al.^4^ and Shaheen et al.^5^. The mean of the calculated R values was used as estimand of R for estimating change values. After estimating the mean and SD of the change where it was not given (imputing the R as described), we pooled together the reported and estimated change values using the previously mentioned inverse variance method. Additionally, this estimation was performed using different R values – as influential analyses - to see how the pooled effect differed.

**3b.** Starting from the estimated data resulted from the 3^rd^ approach (*see above*), we performed an analysis of cough scores on treatment duration dependency, using a multivariate model implemented in the *metafor* package (*rma.mv* function). We assumed the same correlation among timepoints within a study, using the previously mentioned estimated R value (as determined between baseline and after-treatment values). We assumed a compound-symmetry correlation structure between each (categorized) timepoint estimate (i.e. between “true effects” of random models), using the previously mentioned correlation coefficient. To circumvent the issues arising from the estimation of the correlation structure, we supplemented the method with cluster-robust approach test and CI estimation adjustment using clubsandwich method, with small-sample and Satterthwaite approximation implemented in the *clubSandwich* package, using *vcovCR=“CR2”* parameter (according to the recommendations given in *http://wvbauer.com/lib/exe/fetch.php/talks:2021_viechtbauer_dortmund_workflow_ma.html*, chapter Cluster-Robust Inference). We reported these modified results. Additionally, we also ran the model assuming an autoregressive 1 dependency structure (within study, between “true effects”), as influence analysis.

**References**

1. Luo D, Wan X, Liu J, Tong T. Optimally estimating the sample mean from the sample size, median, mid-range, and/or mid-quartile range. *Stat Methods Med Res*. 2018;27(6):1785-1805. doi:10.1177/0962280216669183

2. Shi J, Luo D, Weng H, et al. Optimally estimating the sample standard deviation from the five-number summary. *Res Synth Methods*. 2020;11(5):641-654. doi:10.1002/jrsm.1429

3. Hedges L V. Distribution Theory for Glass’s Estimator of Effect Size and Related Estimators. *J Educ Stat*. 1981;6(2):107. doi:10.2307/1164588

4. Kiljander TO, Junghard O, Beckman O, Lind T. Effect of esomeprazole 40 mg once or twice daily on asthma: A randomized, placebo-controlled study. *Am J Respir Crit Care Med*. 2010;181(10):1042-1048. doi:10.1164/rccm.200910-1537OC

5. Shaheen NJ, Crockett SD, Bright SD, et al. Randomised clinical trial: High-dose acid suppression for chronic cough - A double-blind, placebo-controlled study. *Aliment Pharmacol Ther*. 2011;33(2):225-234. doi:10.1111/j.1365-2036.2010.04511.x
